# Nocturnal Sleep Breathing Patterns in Healthy Adolescents Residing at Very High Altitudes in Bolivia

**DOI:** 10.1111/jsr.70326

**Published:** 2026-03-16

**Authors:** Keaton Patterson, Santiago Ucrós Rodríguez, E. Nicolás Arancibia‐Levit, Fernanda Aliaga Raduan, José Antonio Viruez Soto, Max Gassmann, Silvia Ulrich, Michael Furian, Edith M. Schneider Gasser

**Affiliations:** ^1^ Institute of Veterinary Physiology, Vetsuisse Faculty University of Zürich Zürich Switzerland; ^2^ Department of Pulmonology University Hospital Zürich Zürich Switzerland; ^3^ Universidad de Los Andes Bogotá Colombia; ^4^ Universidad Privada de Santa Cruz de la Sierra (UPSA) Santa Cruz Bolivia; ^5^ Institut Universitaire de Cardiologie et de Pneumologie de Québec (IUCPQ) Laval University Québec Canada; ^6^ Hospital del Norte El Alto Bolivia; ^7^ Zurich Neuroscience Center (ZNZ) University of Zürich and ETH Zürich Zürich Switzerland

**Keywords:** apnea–hypopnea index (AHI), high altitude, hypoxia, oxygen desaturation index (ODI), oxygen saturation (SpO_2_), sleep polygraphy, sleep‐disordered breathing (SDB)

## Abstract

Data on sleep and respiratory patterns among adolescents residing at very high altitude (> 3500 m) remain scarce, and altitude‐related physiological differences may influence these parameters. Studying adolescents at different very high altitudes is crucial, as subtle environmental variations could affect sleep‐related oxygenation and respiratory function. This study aimed to characterise sleep‐related oxygenation and respiratory parameters in healthy adolescents native to two distinct very high‐altitude environments. Overnight sleep polygraphy was performed in 163 healthy adolescents aged 13.5 to < 18 years living in La Paz (3620 m) and El Alto (4060 m), Bolivia. Mean nocturnal oxygen saturation, oxygen desaturation index, and apnea–hypopnea index were assessed alongside subjective sleep quality, morning blood pressure, heart rate, haemoglobin concentration, and Epworth Sleepiness Scale scores. Adolescents at 4060 m had significantly lower mean nocturnal oxygen saturation (84.8% ± 2.2%) compared with those at 3620 m (87.8% ± 1.8%), and a higher oxygen desaturation index (21.2 ± 8.5/h vs. 17.1 ± 9.0/h). The apnea‐hypopnea index did not differ significantly between altitudes (6.2 ± 4.8/h vs. 5.6 ± 4.6/h). At 3620 m, females showed lower oxygen desaturation and apnea‐hypopnea indices compared with males. Despite the more pronounced nocturnal hypoxemia at 4060 m, haemoglobin concentration did not increase, suggesting limited haematological compensation. Subjective sleep quality, blood pressure, and heart rate were similar between both altitude groups. Healthy adolescents living chronically at very high altitude exhibit altitude‐dependent reductions in nocturnal oxygenation and increased desaturation frequency, without evidence of sleep‐disordered breathing. These findings underscore the need for altitude‐specific normative values to support accurate interpretation of sleep studies in high‐altitude populations.

## 1 Introduction

Globally, approximately 81.6 million people reside at altitudes above 2500 m, and an estimated 6.4 million live above 4000 m (Tremblay and Ainslie 2021). Countries such as Bolivia, Peru, and China have some of the largest populations residing at very high altitudes, including Bolivia's El Alto, the world's most populous city located above 4000 m (population 943,558 in 2020 (Casagrande and Horn 2023)).

Sleep is a vital physiological process, particularly during childhood and adolescence, playing a critical role in physical growth, cognitive development, and overall health (Agostini and Centofanti 2021; Goel and Goel 2024). Sleep‐disordered breathing (SDB) in these age groups has been linked to adverse outcomes across multiple domains, including neurocognitive deficits (Beebe 2006a; 2006b; Menzies et al. 2022), cardiovascular alterations (Amin et al. 2002; Kaditis et al. 2016) and metabolic disturbances (Redline et al. 2007). Despite growing advances in sleep medicine, studies focusing on paediatric populations chronically exposed to high altitude environments remain scarce.

Key diagnostic parameters for SDB include the oxygen desaturation index (ODI) and the apnea–hypopnea index (AHI) (Dempsey et al. 2010). Previous studies conducted in South America and Central Asia at elevations between 2500 and 3620 m have reported increased AHI and ODI in healthy infants, children, and adolescents living at high altitude (Duenas‐Meza et al. 2015; Grimm et al. 2023; Hill et al. 2016; Ucros 2021; Ucros, Granados, et al. 2021). However, interpreting sleep data at altitude is challenging due to the combined effects of developmental physiology and chronic hypobaric hypoxia (Duenas‐Meza et al. 2015; Grimm et al. 2023; Hill et al. 2016; Ucros 2021; Ucros, Granados, et al. 2021; Ucros 2015). Applying sea‐level reference values to these populations may result in misclassification, leading to overdiagnosis or underdiagnosis of SDB and subsequent mismanagement (Ucros 2021; Ucros, Granados, et al. 2021).

To date, nocturnal breathing patterns in healthy adolescents permanently residing at very high altitudes (> 3500 m) have not been systematically characterised. Moreover, no study has compared sleep‐related respiratory parameters between two distinct very high‐altitude populations. This cross‐sectional study aimed to evaluate and compare nocturnal blood oxygenation (SpO_2_) and respiratory parameters in healthy native adolescents living in La Paz (3620 m) and El Alto (4060 m), Bolivia. We hypothesized that at very high altitude subtle environmental variations could affect sleep‐related oxygenation and respiratory function, thus adolescents residing at 4060 m would exhibit more pronounced nocturnal hypoxemia and a higher prevalence of SDB compared to those living at 3620 m.

## 2 Methods

### 2.1 Study Design and Participants

This cross‐sectional study was conducted between April and June 2024. Male and female adolescents who were native residents and permanently living in La Paz (3620 m) or El Alto (4060 m), Bolivia, were invited to participate. Participants from each city were assessed in corresponding local facilities.

Exclusion criteria included: age < 13.5 or > 19 years, current use of medication, presence of chronic medical conditions affecting cardiac or pulmonary function, and insufficient pulse oximetry recording time (< 4 h).

The study protocol was approved by the *Gobierno Autónomo Departamental de La Paz Ethics Committee* (GADLP/SEDES/HDN/CB/11/2023). Written informed consent was obtained from all participants and, for minors (< 18 years), from their parents or legal guardians.

### 2.2 Assessments

Upon arrival at the study site, participants completed a structured health questionnaire including demographic data (age, residence), medical history, and the Epworth Sleepiness Scale (ESS) (Johns 1991). Height and weight were measured, and evening haemoglobin (Hb) concentration was determined from a finger‐prick sample using a portable haemoglobin analyser (HemoCue 201+, Viollier).

Participants then underwent overnight attended sleep polygraphy using portable devices (*Samoa*, Löwenstein Medical, Switzerland). Recordings included pulse oximetry, nasal airflow (pressure transducer), body position, and thoracic and abdominal wall movements (inductive plethysmography).

The sleep study was conducted overnight in a field environment adapted from a traditional sleep laboratory, with continuous supervision by trained staff to ensure accurate sensor placement and high‐quality data collection.

The following morning, participants reported subjective sleep parameters, including sleep latency, number of awakenings, total awake time, and overall sleep quality (visual analog scale: 0 = worst imaginable sleep; 100 = best sleep ever). They also completed the Karolinska Sleepiness Scale (Kaida et al. 2006). Resting heart rate and blood pressure were measured three times before participants rose from bed, with the average of the second and third readings being used for analysis.

### 2.3 Sleep and Breathing Analysis

Given the lack of normative paediatric data at very high altitude and the absence of established paediatric reference thresholds for altitude‐related breathing disturbances, nocturnal respiratory events were scored according to the American Academy of Sleep Medicine (AASM) 2012 criteria for adults using *MiniScreen Viewer* software (version 5.23a R1). Apneas were defined as a ≥ 90% reduction in airflow lasting ≥ 10 s, and hypopneas as a ≥ 30% reduction in airflow lasting ≥ 10 s accompanied by ≥ 3% oxygen desaturation. Periodic breathing (PB) was defined as ≥ 3 consecutive central apneas or hypopneas (each ≥ 5 s) separated by ≤ 20 s of normal breathing. The apnea–hypopnea index (AHI) was calculated as the total number of apneas and hypopneas per hour of sleep.

Due to the unique physiological characteristics of high‐altitude populations, thresholds for diagnosing SDB should be interpreted with caution. This highlights the need for altitude‐specific normative data and validation of SDB diagnostic criteria in comparable high‐altitude populations.

Obstructive versus central events were differentiated based on the presence or absence of nasal pressure flattening and thoracoabdominal movement asynchrony. “Evaluation time” represented time in bed after removal of unscorable segments (excessive movement, upright position, or pulse oximetry disconnection). “Nasal flow evaluation time” further excluded periods with unusable nasal cannula data.

Arterial oxygen content (CaO_2_) was calculated as:

$${\mathrm{CaO}}_{2}=\left(1.34^{*}\;{\mathrm{Hb}}^{*}\;{\mathrm{SpO}}_{2}\right)$$

The term PaO_2_ (0.003 X PaO_2_) was omitted from the formula due to a negligible contribution at these altitudes.

### 2.4 Statistical Analysis

Participant characteristics were summarised using descriptive statistics. Continuous variables were expressed as mean ± SD; categorical variables as counts and percentages. Normality of distributions was assessed with the Kolmogorov–Smirnov test.

Group differences between altitudes were examined using independent‐samples *t*‐tests. Two‐way ANOVA models were used to assess the effects of altitude, sex, and their interaction on primary outcomes. Linear regression models were used to estimate mean differences (95% CI) for continuous variables, including altitude (3620 m vs. 4060 m), sex, and altitude*sex interaction as predictors.

Exploratory multivariable linear regression models were constructed to identify independent predictors of mean nocturnal SpO_2_. Predictor variables were selected based on physiological and clinical relevance and included: altitude, sex, age, body mass index (BMI), athlete status (> 8 h of sport training per week), haemoglobin concentration, and percentage of the night spent in periodic breathing.

Statistical analyses were performed using R (RStudio; version 2024.12.0 + 467), and figures were created in GraphPad Prism (version 10.4.2). Statistical significance was defined as *p* < 0.05.

## 3 Results

### 3.1 Participant Characteristics

A total of 174 adolescents were recruited for the study. A total of 11 were excluded: three did not meet the age criteria, five were taking medications, one had a chronic medical condition affecting cardiac or pulmonary function, and two had insufficient pulse oximetry recording time (< 4 h). The final analysed sample included 163 participants, with 78 (50% female) residing at 3620 m and 85 (48.2% female) at 4060 m. An additional five participants with < 4 h of valid nasal cannula recordings were excluded from analyses involving nasal cannula‐derived indices (e.g., AHI) but were included in pulse oximetry‐based parameters analysis and morning evaluations. Participant characteristics by altitude are summarised in Table 1. Sex, age, weight, height, and BMI did not differ significantly between the altitudes.

**TABLE 1:** Participants' characteristics.

Variable	3620 m	4060 m	*p*
*N*, (% female)	78 (50.0%)	85 (48.2%)	0.946
Age, years	15.7 ± 1.1	16.5 ± 1.2	< 0.001
Weight, kg	57.4 ± 9.1	57.2 ± 8.8	0.875
Height, cm	164.1 ± 8.6	164.3 ± 8.6	0.880
BMI, kg/m^2^	21.4 ± 3.0	21.2 ± 2.6	0.635
Athlete^1^, *n* (%)	28 (35.9%)	49 (57.6%)	0.009
Epworth sleepiness scale^2^	9.2 ± 4.5	7.2 ± 3.7	0.004
Haemoglobin, g/dL	16.3 ± 1.5	16.1 ± 2.0	0.411

*Note:* Data is presented as mean ± SD. BMI, body mass index.

^1^ Athlete is defined as participants who complete > 8 h of sport training per week.

^2^ The Epworth Sleepiness Scale is scored from 0 to 24 points, with increasing daytime sleepiness, where a score of 11 or greater represents excessive daytime sleepiness.

Mean age was 15.7 ± 1.1 years in La Paz and 16.5 ± 1.2 years in El Alto. The mean weight was comparable between sites (La Paz: 57.4 ± 9.1 kg; El Alto: 57.2 ± 8.8 kg; *p* = 0.87), as was mean height (164 ± 8.6 cm in both). Mean BMI was 21.4 ± 3.0 kg/m^2^ in La Paz and 21.2 ± 2.6 kg/m^2^ in El Alto. A greater proportion of adolescents in El Alto reported high current sport activity compared with La Paz (57.6% vs. 35.9%; *p* = 0.009).

Daytime sleepiness, assessed using the Epworth Sleepiness Scale, was higher among participants at 3620 m (9.2 ± 4.5) than at 4060 m (7.2 ± 3.7; *p* = 0.004); scores < 11 were considered within the normal range.

### 3.2 Nocturnal Breathing Pattern

Respiratory polygraphy findings are summarised in Table 2. Time in bed, total evaluation time, and valid nasal flow recording time were comparable between 3620 and 4060 m.

**TABLE 2:** Respiratory polygraphy.

Variable	3620 m	4060 m	Mean difference (95% CI), 4060 m versus 3620 m	*p*
Time in bed, min	477 ± 17	475 ± 18	−2 (−7 to 4)	—
Evaluation time^1^, min	433 ± 49	456 ± 37	23 (10 to 36)	—
Nasal flow evaluation time^2^, min	400 ± 93	442 ± 57	42 (19 to 66)	—
Nocturnal SpO_2_ ^3^, %	87.8 ± 1.8	84.8 ± 2.2	−3.0 (−3.6 to −2.4)	< 0.001
T90^3^, %	77.5 ± 24.4	96.5 ± 6.1	18.9 (13.5 to 24.3)	< 0.001
ODI^3^, 1/h	17.1 ± 9	21.2 ± 8.5	4.1 (1.4 to 6.8)	0.003
Total AHI^4^, 1/h	5.6 ± 4.6	6.2 ± 4.8	0.6 (−0.9 to 2.0)	0.466
Obstructive AHI^4^, 1/h	3.1 ± 2.5	3.4 ± 2.5	0.2 (−0.5 to 1.0)	0.545
Obstructive AI^4^, 1/h	0.2 ± 0.5	0.1 ± 0.3	−0.1 (−0.2 to 0.1)	0.433
Obstructive HI^4^, 1/h	2.9 ± 2.4	3.2 ± 2.3	0.3 (−0.4 to 1.0)	0.435
Central AHI^4^, 1/h	2.5 ± 3.4	2.8 ± 4.1	0.3 (−0.9 to 1.5)	0.587
Central AI^4^, 1/h	0.7 ± 1.3	0.6 ± 0.9	−0.1 (−0.5 to 0.2)	0.402
Central HI^4^, 1/h	1.8 ± 2.6	2.3 ± 3.3	0.5 (−0.5 to 1.4)	0.316
Periodic breathing^4^, % evaluation time	0.8 ± 2.2	0.8 ± 2.2	0.0 (−0.7 to 0.6)	0.907
AHI excluding periodic breathing^4^, 1/h	4.6 ± 3.1	5.1 ± 3.1	0.5 (−0.5 to 1.4)	0.351
Nighttime heart rate^3^, bpm	66 ± 10	66 ± 9	0 (−3 to 3)	0.992

*Note:* Data are presented as mean ± SD. Mean differences between groups are shown with 95% confidence intervals and corresponding *P*‐values.

Abbreviations: AHI: apnea‐hypopnea index; AI: apnea index; HI: hypopnea index; ODI: oxygen desaturation index defined as a SpO_2_ desaturation of ≥ 3%; SpO_2_: peripheral oxygen saturation assessed by pulse oximetry; T90: percentage of time in bed spent below 90% SpO_2_.

^1^ Evaluation time represents time in bed after removal of unscorable segments, such as periods with excessive movement, upright position (detected by device's accelerometer), disconnected pulse oximetry or otherwise unscorable data.

^2^ Nasal flow evaluation time is the evaluation time, further excluding periods where nasal cannula data were unusable.

^3^ Calculated on the evaluation time.

^4^ Calculated on nasal flow evaluation time.

**FIGURE 1:**
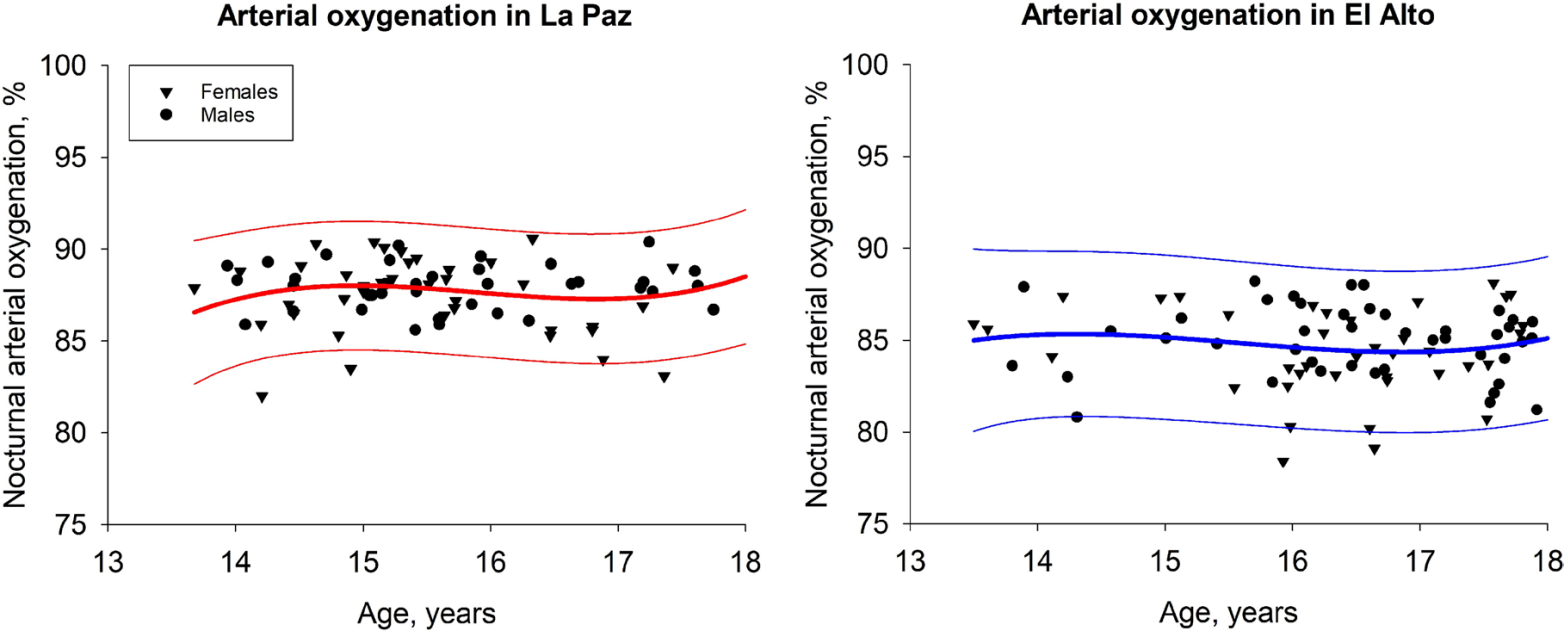
Nocturnal oxygenation by age and sex in adolescents residing at two very high‐altitude locations. Scatter plots show mean nocturnal oxygen saturation (SpO_2_) in healthy adolescents aged 13.5 to < 18 years living in La Paz (3620 m) (left, red) and El Alto (4060 m) (right, blue). Circles represent males and inverted triangles represent females. Solid lines depict fitted regression lines with 95% confidence intervals. No significant associations were found between age and nocturnal oxygenation within each altitude group. Adolescents living at 4060 m exhibited overall lower SpO_2_ compared to those at 3620 m (*p* < 0.001).

**FIGURE 2:**
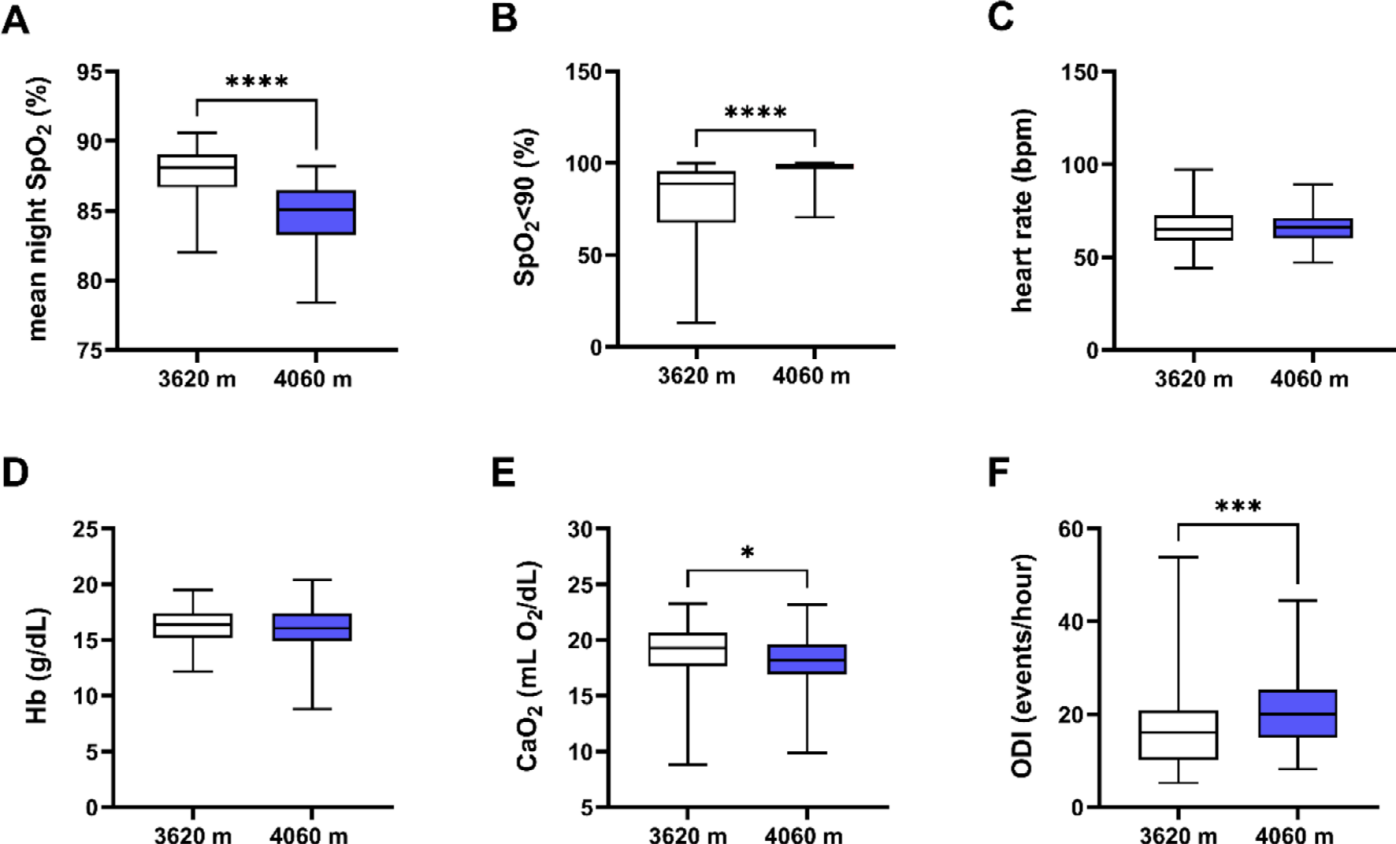
Comparison of mean nocturnal oxygen (SpO_2_, %) T90, heart rate, haemoglobin, arterial oxygen content (CaO_2_), and oxygen desaturation index (ODI) in adolescents residing at very high altitudes. Mean (±SD) nocturnal oxygen saturation (SpO_2_, %) (A), T90 (B), heart rate (C), haemoglobin (Hb) (D), arterial oxygen content (CaO_2_) (E), and oxygen desaturation index (ODI) (F) in adolescents residing at very high altitude and sleeping at 3620 m and 4060 m. *n = 78 (3620 m) and 85 (4060 m)*. Statistical comparisons between groups were performed using a two‐tailed unpaired *t*‐test (A–E) and a Mann–Whitney test (F). *p*‐values are indicated: *p = 0.03, ***p = 0.0006, ****p < 0.0001.

Adolescents living at 4060 m exhibited significantly lower nocturnal arterial oxygen saturation (SpO_2_) compared to those residing at 3620 m (*p* < 0.001). Fitted regression lines with 95% confidence intervals are shown in Figure 1; within each altitude group, no significant associations were observed between age and nocturnal oxygenation. Mean nocturnal SpO_2_ was markedly reduced at 4060 m (84.8% ± 2.2%) relative to 3620 m (87.8% ± 1.8%; *p* < 0.001; Figure 2A). Correspondingly, the proportion of total sleep time spent with SpO_2_ < 90% (T90) was substantially higher at 4060 m (96.5% ± 6.1%) than at 3620 m (77.5% ± 24.4%; *p* < 0.001, Figure 2B). Despite this pronounced nocturnal hypoxemia, mean nocturnal heart rate remained similar between altitudes (66 ± 10 bpm; Figure 2C; Table 2). Notably, haemoglobin concentrations did not differ significantly between groups (3620 m: 16.3 ± 1.5 g/dL; 4060 m: 16.1 ± 2.0 g/dL; *p* = 0.41, Figure 2D; Table 1), indicating a lack of haematological compensation for the greater hypoxic burden in El Alto. Consequently, adolescents at 4060 m displayed a lower calculated arterial oxygen content (CaO_2_), (3620 m: 19.19 ± 1.91 mL O_2_/dL; 4060 m: 18.27 ± 2.36 mL O_2_/dL; mean difference of −0.91 mL O_2_/dL; 95% CI, −1.59 to −0.24; *p* = 0.008; unpaired *t*‐test; Figure 2E), suggesting limited physiological adaptation to chronic nocturnal desaturation at this extreme altitude.

The oxygen desaturation index (ODI) was significantly higher in adolescents residing at 4060 m (21.2 ± 8.5 events/h) compared to those at 3620 m (17.1 ± 9.0 events/h; *p* = 0.0006; Table 2; Figure 1F), indicating more frequent nocturnal desaturation events at higher altitude. In contrast, the apnea–hypopnea index (AHI) did not differ significantly between altitude groups (3620 m: 5.6 ± 4.6 events/h; 4060 m: 6.2 ± 4.8 events/h; *p* = 0.466). Similarly, neither obstructive nor central AHI varied between the two altitudes. Obstructive events accounted for the majority of total respiratory events (55.4% in La Paz; 54.8% in El Alto), with hypopneas representing the predominant component of these obstructive events (93.5% and 94.1%, respectively). Despite the greater nocturnal hypoxemia and higher ODI observed at 4060 m, the stable AHI across altitudes suggests that oxygen desaturation, rather than increased event frequency, is the main driver of nocturnal hypoxic stress at very high altitude.

**FIGURE 3:**
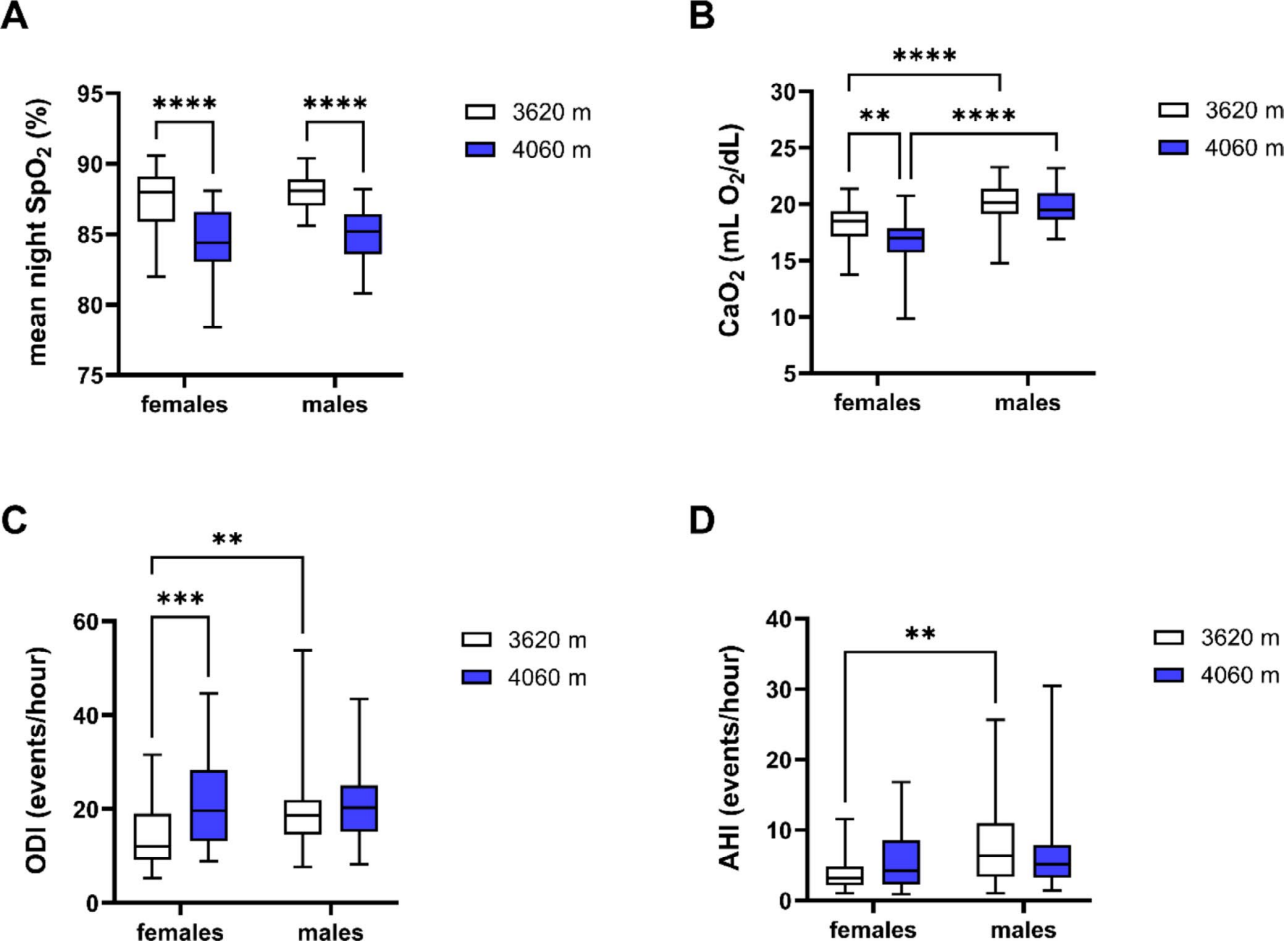
Comparison of mean nocturnal oxygen (SpO_2_), arterial oxygen content (CaO_2_), oxygen desaturation index (ODI), and apnea‐hypopnea index (AHI) by altitude and sex. Mean (±SD) nocturnal oxygen saturation (SpO_2_, %) (A), arterial oxygen content (CaO_2_) (B), oxygen desaturation index (ODI) (C), and apnea–hypopnea index (AHI) (D) in female and male adolescents residing at very high altitude and sleeping at 3620 m and 4060 m. Comparisons were performed between sexes (female vs. male) and altitudes (3620 m vs. 4060 m). *n = 39 females and 39 males (3620 m)*, *and 41 females*, *44 males (4060 m)*. Statistical analyses were conducted using two‐way ANOVA, with significance levels indicated as: **p < 0.01, ***p < 0.001, ****p < 0.0001.

Subgroup analyses by sex revealed no significant differences in mean nocturnal SpO_2_ (Figure 3A). CaO_2_ was significantly lower in females living at 4060 m (mean difference of −2.372 mL O_2_/dL; 95% CI, −2.935 to −1.809; *p* < 0.0001, Figure 3B).

Females living at 3620 m had significantly lower ODI than males (14.0 ± 6.8 vs. 20.2 ± 9.9 events/h; mean difference of −6.3 events/h; 95% CI, −10.1 to −2.5; *p* < 0.001, Figure 3C). Females also showed lower AHI (4.0 ± 2.7 vs. 7.5 ± 5.6 events/h; mean difference of −3.5; 95% CI, −5.6 to −1.4; *p* < 0.01, Figure 3D), driven primarily by lower obstructive AHI (2.3 ± 1.6 vs. 4.1 ± 3.0 events/h; mean difference = −1.8; 95% CI, −3.0 to −0.7; Table S1) compared with males at 3620 m. These sex differences were not observed at 4060 m, where male and female participants exhibited similar ODI and AHI (Table S1; Figure 3C,D). The increase in ODI with altitude was significantly greater among females than males, with a sex‐related mean difference in altitude effect of 7.5 events/h (95% CI, 2.3 to 12.8; Table S1, Figure 3C).

### 3.3 Subjective Sleep Quality and Clinical Examination in the Morning

Morning evaluation metrics indicated similar subjective sleep quality ratings between participants residing at 3620 and 4060 m. Adolescents at 4060 m reported a slightly shorter sleep latency (26 ± 26 min vs. 38 ± 33 min, *p* = 0.01; Table 3). No differences were observed between altitude groups in the number of self‐reported awakenings at night (2 ± 1), estimated awake time during the night (22 ± 31 min vs. 16 ± 23 min, *p* = 0.17), Karolinska Sleepiness Scale scores, or overall subjective sleep quality (Table 3). Morning clinical parameters were also comparable between altitudes. Heart rate and systolic blood pressure did not differ, whereas diastolic blood pressure was slightly lower at 4060 m compared with 3620 m (Table 3).

**TABLE 3:** Subjective sleep quality and clinical examination in the morning.

Variable	3620 m	4060 m	Mean difference (95% CI), 4060 m versus 3620 m	*p*
Subjective time until falling asleep, min	38 ± 33	26 ± 26	−12 (−21 to −3)	0.010
Number of awakenings at night, *n*	2 ± 1	2 ± 1	0 (0 to 1)	0.174
Estimated awake time at night, min	22 ± 31	16 ± 23	−6 (−14 to 3)	0.173
Karolinska sleepiness scale^1^	5 ± 2	4 ± 2	−1 (−1 to 0)	0.072
Subjective sleep quality^2^, %	72 ± 17	70 ± 18	−2 (−7 to 4)	0.586
Heart rate, bpm	67 ± 12	68 ± 10	1 (−2 to 5)	0.541
Systolic blood pressure, mmHg	106 ± 8	104 ± 8	−2 (−4 to 1)	0.216
Diastolic blood pressure, mmHg	65 ± 6	62 ± 6	−2 (−4 to 0)	0.032

*Note:* Data are presented as mean ± SD. Mean differences between altitudes are shown with 95% confidence intervals and corresponding *P*‐values.

^1^ Karolinska sleepiness scale is a 9‐point scale used to estimate subjective sleepiness at a particular time, scored from 1 to 9 points with increasing sleepiness.

^2^ Subjective sleep quality was assessed by a 100‐mm visual analog scale ranging from 0 “worst imaginable sleep” to 100 “best sleep ever”. The score was transposed to a 0% to 100% score, where higher scores indicate better sleep quality.

Sex‐related analyses of height, weight, heart rate, haemoglobin, diastolic and systolic blood pressure showed no significant sex‐by‐altitude interaction effects (Table S2; Figure S1). At 3620 m, females exhibited higher heart rates than males (72 ± 12 vs. 62 ± 11 bpm; mean difference of 10 bpm; 95% CI, 5 to 15; Figure S1C).

No difference in diastolic blood pressure among sexes and altitude was observed (Figure S1E). However, females had lower systolic blood pressure than males at both altitudes (3620 m: 101 ± 7 vs. 110 ± 7 mmHg; mean difference of −9 mmHg; 95% CI, −12 to −6; 4060 m: 99 ± 7 vs. 108 ± 6 mmHg; mean difference of −9 mmHg; 95% CI, −16 to −2, Figure S1F).

### 3.4 Predictors of Nocturnal SpO_2_ Based on Multivariable Linear Regression Models

Exploratory multivariable linear regression analyses identified several independent predictors of nocturnal SpO_2_ (Table S3). Residence at 4060 m, sex, higher oxygen desaturation index (ODI), and lower periodic breathing were independently associated with reduced nocturnal SpO_2_ (Table S3). However, other variables, including age (pubertal maturation), body mass index, physical fitness, and haemoglobin concentration were not significantly associated with nocturnal SpO_2_ (Table S3).

## 4 Discussion

This study is the first to investigate sleep polygraphy parameters in healthy, native Bolivian adolescents permanently residing at two adjacent high‐altitude locations, La Paz (3620 m) and El Alto (4060 m), providing unique data from adolescents permanently residing above 4000 m. Our findings demonstrate substantial altitude‐related differences in arterial oxygen content (CaO_2_) and oxygen desaturation index (ODI) between these very high‐altitude settings, whereas obstructive and central apnea–hypopnea indices (AHI) did not differ. Heart rate, clinical examination findings, and subjective sleep quality assessed the following morning were comparable between sites and not clinically different from expected values reported at low altitude. These findings suggest that residence at 3620 or 4060 m minimally impacts clinical and subjective morning measures in healthy adolescents. Exploratory analyses indicated that females were more prone to nocturnal hypoxemia, whereas males exhibited higher ODI.

The mean AHI values observed (5.6 ± 4.6 for La Paz and 6.2 ± 4.8 for El Alto) were higher than those reported for low‐altitude adolescents (AHI < 1) and young adults (AHI < 1.4) (Boulos et al. 2019; Uliel et al. 2004). Obstructive AHI values in our cohort (3.1 events/h) exceeded those previously reported in Bolivian children (2.1 events/h), while central apnea indices were comparable (0.7 events/h) (Hill et al. 2016). The higher obstructive AHI in our study likely reflects differences in scoring criteria: we applied AASM 2012 definitions (≥ 30% airflow reduction with ≥ 3% desaturation), whereas Hill et al. used a ≥ 50% reduction threshold (Hill et al. 2016; Berry et al. 2012). Consistent with prior reports from Andean populations, we observed a predominance of obstructive hypopneas at both altitudes (Duenas‐Meza et al. 2015; Grimm et al. 2023; Hill et al. 2016). Hill et al. described frequent obstructive events in Bolivian children aged 7–16 y native to 3650 m, with few central events (Duenas‐Meza et al. 2015; Hill et al. 2016; Ucros 2021). Similarly, studies in Colombian children living at 2560–2640 m found that obstructive events accounted for most of the AHI (Duenas‐Meza et al. 2015; Ucros 2021). In contrast, studies of high‐altitude children in the Kyrgyz Republic and preschoolers in Denver (1600 m) attributed elevated AHI primarily to central apneas (Grimm et al. 2023; Burg et al. 2013). These divergent patterns may reflect population‐specific adaptations or developmental differences and warrant further investigation.

With respect to oxygen desaturation, ODI increased at 4060 m (21.2 ± 8.5 events/h) compared with 3620 m (17.1 ± 9.0 events/h), accompanied by a reduction in mean nocturnal SpO_2_ (from 87.8% to 84.8%) and a rise in T90 (from 77.5% to 96.5%). Despite these differences, AHI remained stable between altitudes (5.6 vs. 6.2 events/h), suggesting that the higher ODI reflects increased variability in oxygen saturation due to subtle ventilatory fluctuations not meeting the hypopnea or apnea threshold. At 3620 m, females exhibited lower ODI, AHI, and obstructive AHI than males; however, these sex‐related differences were absent at 4060 m. This may indicate altitude‐dependent modulation of sex effects, highlighting the need for further research on sex‐specific adaptation to chronic hypoxia. In sojourner populations, females exhibit less periodic breathing and fewer hypopneas during high‐altitude exposure (Caravita et al. 2015; Lombardi et al. 2013; Patrician et al. 2024; Torlasco et al. 2020). Whether similar patterns persist among native highlanders remains unclear.

Population composition may also contribute to these findings. The El Alto cohort is predominantly Aymara, a group with a long evolutionary history of residence at a very high altitude, whereas the La Paz population is more heterogeneous, with greater Hispanic admixture. Genetic background likely influences ventilatory control and hematologic responses to chronic hypoxia and should be considered when interpreting these results. Future studies should incorporate genetic ancestry, cultural, and lifestyle factors to better contextualise nocturnal breathing phenotypes. Comparison with low‐altitude data underscores these adaptations. Hill et al. reported markedly lower respiratory indices in low‐altitude Bolivian children (obstructive AHI = 0.6; central AI = 0.3; ODI = 0.6 events/h at 500 m) (Hill et al. 2016). Similar findings have been observed in other low‐altitude paediatric populations (Grimm et al. 2023; Verhulst et al. 2007). These data emphasise that applying low‐altitude normative thresholds to high‐altitude residents could lead to overdiagnosis of SDB (Ucros 2021; Ucros, Granados, et al. 2021). Moreover, although adolescents show relatively high numbers of self‐reported awakenings and wake after sleep onset (WASO) compared to children (Colrain and Baker 2011), data from adolescents at sea level indicate no significant difference in WASO, averaging around 24 min (Hill et al. 2016). In contrast, studies in healthy adult highlanders (mean age 39 years) report higher WASO values, approximately 72 min, suggesting that wakefulness after sleep onset increases with age and/or chronic high‐altitude exposure.

Interestingly, daytime sleepiness, assessed by the Epworth Sleepiness Scale (ESS), was higher at 3620 m than at 4060 m. Using the conventional cutoff (> 10), 41% of participants in La Paz met criteria for excessive daytime sleepiness (EDS), compared with 17.6% in El Alto. Reference data from 3871 adolescents at sea level (mean age ≈16.8 y) reported EDS prevalence of 14.9% in males and 18.2% in females (Joo et al. 2005), suggesting that the prevalence in El Alto is comparable to sea‐level norms, whereas La Paz adolescents show an elevated burden of EDS. The difference in ESS scores between the two cities was not correlated with higher physical fitness or age in El Alto. However, a higher proportion of individuals in El Alto with Aymara ancestry, who have longer generational exposure to high altitude and greater physiological resilience to hypoxia, may contribute to the observed pattern. Notably, age, athletic activity, and ethnicity did not have a significant impact on nocturnal SpO_2_ saturation or breathing patterns.

Distinct high‐altitude populations exhibit different adaptive mechanisms. Andean populations, unlike Tibetans, show greater haemoglobin increases with altitude (Beall et al. 1998; Bigham et al. 2013; Mairbaurl et al. 2020). In our cohort, mean haemoglobin concentration (16.2 g/dL) aligns with prior reports from Andean populations (Gassmann et al. 2019; Leon‐Velarde et al. 2000; Wang 2016). For instance, Peruvian children at 4355 m demonstrated haemoglobin levels of 16.1 g/dL in males and 15.6 g/dL in females, rising with age (Leon‐Velarde et al. 2000). Despite greater hypoxia and older age at 4060 m, we found no altitude‐related increase in haemoglobin, and haemoglobin was not associated with mean SpO_2_ in multivariable analyses. This contrasts with findings in Andean adults showing that erythrocytosis correlates with lower nocturnal SpO_2_ (Spicuzza et al. 2004).

Estimated CaO_2_ was 19.19 mL O_2_/dL at 3620 m and 18.27 mL O_2_/dL at 4060 m, indicating that higher altitude was not accompanied by further compensatory oxygen‐carrying capacity. For reference, a healthy adolescent at low altitude (haemoglobin = 14 g/dL; SpO_2_ = 98%) would be expected to have a CaO_2_ of approximately 18.4 mL O_2_/dL (Romeo et al. 2009), similar to the values in our cohort. Thus, despite moderate nocturnal desaturation, haemoglobin elevation at high altitude appears sufficient to maintain arterial oxygen content within normal physiological limits.

Finally, although adolescents at 4060 m exhibited lower nocturnal SpO_2_ and higher ODI, they did not display clinical or subjective evidence of physiological stress. Morning heart rate, blood pressure, and sleepiness were similar between altitudes, supporting the interpretation that the respiratory differences observed represent adaptive rather than pathological responses.

Several limitations should be acknowledged. Full polysomnography was not feasible in the field, limiting assessment of sleep architecture and arousal‐related scoring. Recordings were restricted to a single night, so first‐night effects cannot be excluded. Nevertheless, the use of standardised equipment and identical protocols at both altitudes minimises systematic bias and strengthens internal validity.

## 5 Conclusion

This study provides novel data on nocturnal respiratory physiology in healthy adolescents, an underrepresented group in high‐altitude research. We present the first detailed comparison of nocturnal oxygenation and breathing patterns among Bolivian adolescents permanently residing at two very high altitudes, 3620 and 4060 m. Even modest differences in elevation were associated with significant reductions in nocturnal SpO_2_ and increased desaturation burden, whereas the frequency of respiratory events and subjective sleep quality remained comparable between altitudes. Approximately 55% of apnea–hypopnea index (AHI) events were obstructive in nature.

These results establish robust reference values for nocturnal respiratory parameters in healthy adolescents chronically residing at very high altitude. Importantly, the higher AHI values observed compared to low‐altitude populations using the AASM standards suggest that diagnostic thresholds derived from sea‐level and adult data may overestimate the prevalence of SDB in high‐altitude and adolescent residents. Therefore, alternative paediatric scoring criteria might yield different absolute indices and clinical interpretations in this population.

## Author Contributions

Edith M. Schneider Gasser and Santiago Ucrós Rodríguez conceived and designed the study. Edith M. Schneider Gasser and Nicolás Arancibia‐Levit recruited participants. José Antonio Viruez Soto contributed to obtaining ethical permits and organising study facilities. Keaton Patterson, Fernanda Aliaga Raduan, and Edith M. Schneider Gasser collected the polygraphy and physiological data. Keaton Patterson, Nicolás Arancibia‐Levit, Michael Furian, and Edith M. Schneider Gasser performed data analysis and statistical evaluation. Santiago Ucrós Rodríguez, Max Gassmann, and Silvia Ulrich contributed to data interpretation and drafting of the manuscript. Keaton Patterson, Edith M. Schneider Gasser, and Michael Furian wrote the first draft of the paper. All authors critically reviewed the manuscript, contributed to revisions, and approved the final version for submission.

## Funding

This work was supported by the Stiftung für wissenschaftliche Forschung an der Universität Zürich (STWF‐23‐010).

## Conflicts of Interest

The authors declare no conflicts of interest.

## Supporting information


**Data S1:** Supporting Information.

## Data Availability

All relevant data are within the paper and its Supporting Information files.
