# Supplementary material for: Nocturnal Sleep Breathing Patterns in Healthy Adolescents Residing at Very High Altitudes in Bolivia
Source: J Sleep Res. 2026 Mar 16;35(4):e70326. doi: 10.1111/jsr.70326 (PMC13357876; doi:10.1111/jsr.70326)

**Supplement to**

**Nocturnal Sleep Breathing Patterns in Healthy Adolescents Living at Very High Altitudes in Bolivia**

*Keaton Patterson¹˒², Santiago Ucrós Rodríguez³, E. Nicolás Arancibia-Levit⁴, Fernanda Aliaga Raduan⁵, José Antonio Viruez Soto⁶, Max Gassmann¹, Silvia Ulrich², Michael Furian², and Edith M. Schneider Gasser¹˒²˒⁴˒⁷*

^1^ Institute of Veterinary Physiology, Vetsuisse Faculty, University of Zürich, Zürich, Switzerland

^2^ Department of Pulmonology, University Hospital Zürich, Zürich, Switzerland

³ Universidad de Los Andes, Bogotá, Colombia

⁴ Universidad Privada de Santa Cruz de la Sierra (UPSA), Santa Cruz, Bolivia

⁵ Institut Universitaire de Cardiologie et de Pneumologie de Québec (IUCPQ), Laval University, Québec, Canada

⁶ Hospital del Norte, El Alto, Bolivia

⁷ Zurich Neuroscience Center (ZNZ), University of Zürich and ETH Zürich, Zürich, Switzerland

**Correspondence:***Edith M. Schneider Gasser*: edith.schneidergasser@uzh.ch; [edith.schneidergasser@usz.ch](mailto:edith.schneidergasser@usz.ch)

*Michael Furian*: [michael.furian@usz.ch](mailto:michael.furian@usz.ch)

| **Table S1: Sex-related differences in respiratory polygraphy parameters** | | | | | | | | | |
| --- | --- | --- | --- | --- | --- | --- | --- | --- | --- |
| **Variable** | **3620 m** | | | **4060 m** | | | **Altitude effect in females (95% CI)** | **Altitude effect in males (95% CI)** | **Sex-related difference in altitude effect (95% CI)** |
|  | **Females** | **Males** | **Sex-related difference  (95% CI)** | **Females** | **Males** | **Sex-related difference  (95% CI)** |  |  |  |
| Time in bed, min | 477 ± 17 | 477 ± 18 | -1 (-9 to 7) | 472 ± 20 | 478 ± 16 | -6 (-25 to 13) | -4 (-23 to 14) | 1 (-7 to 8) | -5 (-16 to 6) |
| Evaluation time^1^, min | 434 ± 42 | 433 ± 56 | 1 (-18 to 21) | 456 ± 32 | 456 ± 41 | 0 (-46 to 46) | 22 (-23 to 68) | 24 (5 to 43) | -1 (-28 to 26) |
| Nasal flow evaluation time^2^, min | 422 ± 54 | 379 ± 117 | 43 (9 to 77) | 439 ± 56 | 445 ± 58 | -6 (-86 to 74) | 18 (-62 to 97) | 67 (34 to 99) | -49 (-96 to -3) |
| Nocturnal SpO_2_ ^3^, % | 87.5 ± 2.1 | 88.0 ± 1.2 | -0.5 (-1.4 to 0.4) | 84.4 ± 2.4 | 85.1 ± 1.9 | -0.6 (-2.8 to 1.5) | -**3.1 (-5.2 to -1.0)** | **-3.0 (-3.8 to -2.1)** | -0.1 (-1.4 to 1.1) |
| T90 ^3^, % | 77.1 ± 27.5 | 78.0 ± 21.2 | -0.9 (-8.8 to 6.9) | 96.2 ± 6.7 | 96.7 ± 5.6 | -0.5 (-19.2 to 18.2) | **19.1 (0.6 to 37.6)** | **18.7 (11.1 to 26.3)** | 0.4 (-10.5 to 11.3) |
| ODI ^3^, 1/h | **14.0 ± 6.8** | **20.2 ± 9.9** | **-6.3 (-10.1 to -2.5)** | 21.8 ± 10.0 | 20.6 ± 6.9 | 1.3 (-7.8 to 10.3) | 7.9 (-1.1 to 16.8) | 0.4 (-3.3 to 4.0) | **7.5 (2.3 to 12.8)** |
| Total AHI ^4^, 1/h | **4.0 ± 2.7** | **7.5 ± 5.6** | **-3.5 (-5.6 to -1.4)** | 5.8 ± 4.3 | 6.6 ± 5.3 | -0.8 (-5.8 to 4.2) | 1.8 (-3.2 to 6.8) | -0.9 (-3.0 to 1.2) | 2.7 (-0.2 to 5.6) |
| Obstructive AHI ^4^, 1/h | **2.3 ± 1.6** | **4.1 ± 3.0** | **-1.8 (-3.0 to -0.7)** | 3.0 ± 2.5 | 3.7 ± 2.4 | -0.6 (-3.3 to 2.0) | 0.8 (-1.8 to 3.4) | -0.4 (-1.5 to 0.7) | 1.2 (-0.3 to 2.7) |
| Obstructive AI ^4^, 1/h | 0.1 ± 0.2 | 0.3 ± 0.7 | -0.2 (-0.4 to 0.0) | 0.2 ± 0.4 | 0.1 ± 0.2 | 0.0 (-0.4 to 0.5) | 0.1 (-0.4 to 0.5) | -0.2 (-0.4 to 0.0) | 0.2 (0.0 to 0.5) |
| Obstructive HI ^4^, 1/h | **2.2 ± 1.6** | **3.8 ± 2.8** | **-1.6 (-2.7 to -0.6)** | 2.9 ± 2.3 | 3.6 ± 2.3 | -0.7 (-3.2 to 1.8) | 0.7 (-1.8 to 3.2) | -0.2 (-1.3 to 0.8) | 1.0 (-0.5 to 2.4) |
| Central AHI ^4^, 1/h | 1.7 ± 2.0 | 3.4 ± 4.3 | -1.7 (-3.4 to 0.1) | 2.8 ± 3.4 | 2.9 ± 4.6 | -0.2 (-4.2 to 3.9) | 1.0 (-3.0 to 5.1) | -0.5 (-2.1 to 1.2) | 1.5 (-0.9 to 3.8) |
| Central AI ^4^, 1/h | 0.5 ± 0.6 | 1.0 ± 1.7 | -0.5 (-1.0 to 0.0) | 0.5 ± 0.9 | 0.6 ± 1.0 | -0.1 (-1.3 to 1.2) | 0.0 (-1.2 to 1.2) | -0.4 (-0.9 to 0.1) | 0.4 (-0.3 to 1.1) |
| Central HI ^4^, 1/h | 1.2 ± 1.6 | 2.4 ± 3.2 | -1.2 (-2.5 to 0.2) | 2.2 ± 2.8 | 2.3 ± 3.7 | -0.1 (-3.3 to 3.1) | 1.0 (-2.2 to 4.2) | -0.1 (-1.4 to 1.2) | 1.1 (-0.8 to 2.9) |
| Periodic breathing ^4^, % evaluation time | 0.5 ± 1.1 | 1.2 ± 3.0 | -0.7 (-1.7 to 0.3) | 0.7 ± 1.4 | 0.8 ± 2.7 | 0.0 (-2.4 to 2.4) | 0.3 (-2.1 to 2.7) | -0.4 (-1.4 to 0.6) | 0.7 (-0.7 to 2.1) |
| Nighttime heart rate ^3^, bpm | **70 ± 10** | **62 ± 9** | **8 (4 to 12)** | 69 ± 8 | 63 ± 9 | 6 (-3 to 16) | -1 (-10 to 9) | 1 (-3 to 5) | -1 (-7 to 4) |
| Data are presented as mean ± SD. Mean differences between groups are shown with 95% confidence intervals. Significant differences are highlighted in bold. ^1^Evaluation time represents time in bed after removal of unscorable segments, such as periods with excessive movement, upright position (detected by device’s accelerometer), disconnected pulse oximetry or otherwise unscorable data; ^2^Nasal flow evaluation time is the evaluation time, further excluding periods where nasal cannula data were unusable; ^3^Calculated on the evaluation time; ^4^Calculated on nasal flow evaluation time. SpO_2_, peripheral oxygen saturation assessed by pulse oximetry; T90%, percentage of time in bed spent below 90% SpO_2_; ODI, oxygen desaturation index defined as a SpO_2_ desaturation of ≥3%; AHI, apnea-hypopnea index; AI, apnea index; HI, hypopnea index | | | | | | | | | |

| **Table S2: Sex-related differences in subjective sleep quality and clinical examination in the morning** | | | | | | | | | |
| --- | --- | --- | --- | --- | --- | --- | --- | --- | --- |
| **Variable** | **3620 m** | | | **4060 m** | | | **Altitude effect in women (95% CI)** | **Altitude effect in men (95% CI)** | **Sex-related difference in altitude effect (95% CI)** |
|  | **Female** | **Male** | **Sex-related difference (95% CI)** | **Female** | **Male** | **Sex-related difference (95% CI)** |  |  |  |
| Subjective time until falling asleep, min | 42 ± 40 | 34 ± 24 | 8 (-6 to 21) | 22 ± 16 | 30 ± 32 | -8 (-39 to 24) | -20 (-51 to 12) | -5 (-17 to 8) | -15 (-34 to 3) |
| Number of awakenings at night, n | 2 ± 1 | 2 ± 2 | 0 (-1 to 1) | 1 ± 1 | 2 ± 1 | -1 (-2 to 1) | 0 (-2 to 1) | 1 (0 to 1) | -1 (-2 to 0) |
| Estimated awake time at night, min | 16 ± 27 | 27 ± 33 | -10 (-22 to 2) | 11 ± 15 | 20 ± 28 | -10 (-38 to 19) | -6 (-34 to 23) | -6 (-18 to 5) | 1 (-16 to 17) |
| Karolinska sleepiness scale ^1^ | 5 ± 2 | 4 ± 2 | 1 (0 to 2) | 4 ± 2 | 4 ± 2 | 0 (-2 to 2) | -1 (-3 to 1) | 0 (-1 to 1) | -1 (-2 to 0) |
| Subjective sleep quality ^2^, % | 74.4 ± 15 | 69.4 ± 19 | 4.9 (-2.8 to 12.7) | 74.7 ± 15 | 66.3 ± 19 | 8.4 (-10.1 to 26.9) | 0.3 (-17.9 to 18.6) | -3.1 (-10.7 to 4.5) | 3.4 (-7.3 to 14.2) |
| Heart rate, bpm | **72 ± 12** | **62 ± 11** | **10 (5 to 15)** | 71 ± 10 | 65 ± 9 | 6 (-5 to 17) | -1 (-12 to 10) | 3 (-2 to 8) | -4 (-11 to 2) |
| Systolic blood pressure, mmHg | **101 ± 7** | **110 ± 7** | **-9 (-12 to -6)** | **99 ± 7** | **108 ± 6** | **-9 (-16 to -2)** | -2 (-9 to 5) | -1 (-4 to 2) | 0 (-5 to 4) |
| Diastolic blood pressure, mmHg | 65 ± 6 | 65 ± 7 | 0 (-3 to 3) | 62 ± 7 | 63 ± 7 | -1 (-8 to 6) | -3 (-10 to 4) | -2 (-5 to 1) | -1 (-5 to 3) |
| Data are presented as mean ± SD. Mean differences between altitudes are shown with 95% confidence intervals. ^1^ Karolinska sleepiness scale is a 9-point scale used to estimate subjective sleepiness at a particular time, scored from 1 to 9 points with increasing sleepiness. ^2^ Subjective sleep quality was assessed by a 100-mm visual analog scale ranging from 0 “worst imaginable sleep” to 100 “best sleep ever”. The score was transposed to a 0 to 100% score, where higher scores indicate better sleep quality. | | | | | | | | | |

| **Table S3: Predictors for nocturnal SpO_2_. Results from a multivariable linear regression analysis** | | | | |
| --- | --- | --- | --- | --- |
| **Predictor** | **Coefficient** | **95% CI** | **SE** | **P-Value** |
| 4060 m vs 3620 m | -2.48 | -3.17 to -1.79 | 0.35 | <0.001 |
| Female vs Male | -0.83 | -1.61 to -0.06 | 0.39 | 0.035 |
| Age, years | 0.02 | -0.24 to 0.28 | 0.13 | 0.855 |
| Body mass index, kg/m^2^ | -0.04 | -0.16 to 0.08 | 0.06 | 0.523 |
| Athletic status | -0.12 | -0.77 to 0.52 | 0.33 | 0.705 |
| Hemoglobin, g/dL | -0.01 | -0.22 to 0.20 | 0.10 | 0.925 |
| ODI, 1/h^1^ | -0.11 | -0.16 to -0.07 | 0.02 | <0.001 |
| Periodic breathing, %^2^ | 0.24 | 0.07 to 0.41 | 0.09 | 0.005 |
| Intercept | 89.72 | 84.26 to 95.18 | 2.76 | <0.001 |
| Data are presented as regression coefficients with 95% confidence intervals, standard errors (SE), and corresponding P-values. ^1^ Calculated on the evaluation time; ^2^ Calculated on the nasal flow evaluation time. SpO_2_, peripheral oxygen saturation assessed by finger oximetry; ODI, oxygen desaturation index defined as SpO_2_ desaturations of >3%. Athletic status is defined as participants who complete > 8 hours of sport per week. | | | | |

**Figure S1.** Mean (±SD) values of height (A), weight (B), heart rate (C), hemoglobin (Hb) (D), diastolic blood pressure (E), and systemic blood pressure (F) in female and male adolescents residing at very high altitude and sleeping at 3620 m and 4060 m. Comparisons were performed between sexes (female vs. male) and altitudes (3620 m vs. 4060 m). Error bars represent standard deviations. *n = 39 females and 39 males (3620m), and 41 females, 44 males (4060m).* Statistical analyses were conducted using two-way ANOVA, with significance levels indicated as ***p < 0.05, **p < 0.01, *****p < 0.001, ****p < 0.0001.


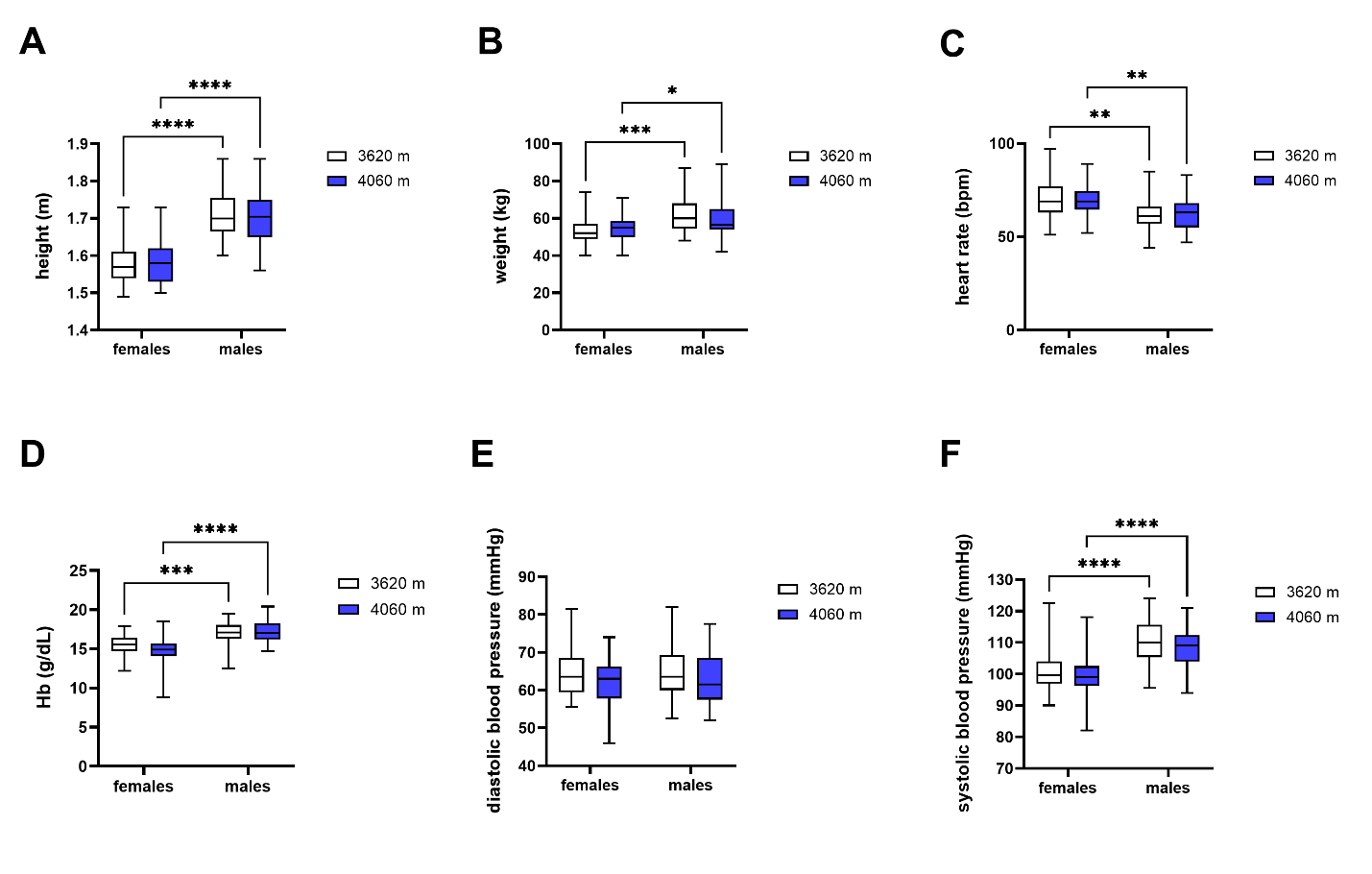

Supplement: Supplementary file 1 — Data S1: Supporting Information. [file JSR-35-e70326-s001.docx]
